# Adiponectin receptor 1 could explain the sex differences in molecular basis of cognitive improvements induced by exercise training in type 2 diabetic rats

**DOI:** 10.1038/s41598-023-43519-7

**Published:** 2023-09-27

**Authors:** Mohammad Amin Rajizadeh, Amirhossein Moslemizadeh, Mahdieh Sadat Hosseini, Forouzan Rafiei, Zahra Soltani, Kayvan Khoramipour

**Affiliations:** 1 Student Research Committee, Faculty of Medicine, Kerman University of Medical Sciences, Kerman, Iran; 2 Neuroscience Research Center, Institute of Neuropharmacology, Kerman University of Medical Sciences, Kerman, Iran; 3 Physiology Research Center, Institute of Neuropharmacology, Kerman University of Medical Sciences, Kerman, Iran; 4 Department of Immunology, Tehran University of Medical Sciences, Tehran, Iran; 5 Health Solutions, College of (CHS), Arizona State University, Phoenix, AZ USA; 6 Endocrinology and Metabolism Research Center, Institute of Basic and Clinical Physiology Sciences, Afzalipour Faculty of Medicine, Kerman University of Medical Sciences, Kerman, Iran

**Keywords:** Molecular biology, Neuroscience, Physiology, Endocrinology, Molecular medicine

## Abstract

Adipokines dysregulation, the main reason for cognitive impairments (CI) induced by diabetes, shows a sex-dependent pattern inherently and in response to exercise. This study aimed to compare the attenuating effect of 8-week high intensity-interval training (HIIT) on type 2 diabetes (T2D)-induced CI between male and female rats with a special focus on adiponectin and leptin. 28 male & 28 female Wistar rats with an average age of 8 weeks were randomly assigned into four groups: control (Con), exercise (EX), Diabetes (T2D), and Type 2 diabetes + exercise (T2D + Ex). Rats in EX and T2D + EX groups performed HIIT for eight weeks (80–100% Vmax, 4–10 intervals). T2D was induced by 2 months of a high-fat diet and a single dose of STZ (35 mg/kg) administration. Leptin and adiponectin levels in serum were measured along with hippocampal expression of leptin and adiponectin receptors, AMP-activated protein kinase (AMPK), dephosphorylated glycogen synthase kinase-3 beta (Dep-GSK3β), Tau, and beta-amyloid (Aβ). Homeostasis model assessments (HOMAs) and quantitative insulin-sensitivity check index (QUICKI) indices were calculated. Our results showed that following T2D, serum levels of APN, and hippocampal levels of adiponectin receptor 1 (APNR1) were higher and HOMA-IR was lower in female than male rats (P < 0.05). However, after 8 weeks of HIIT, hippocampal levels of APNR1 and AMPK as well as QUICKI were lower and hippocampal levels of GSK, Tau, and Aβ were higher in females compared to male rats (P < 0.05). While the risk of CI following T2D was more in male than female rats HIIT showed a more ameliorating effect in male animals with APN1 as the main player.

## Introduction

Problems with a person’s ability to think, learn, remember, use judgment, and make decisions are called cognitive impairment (CI)^1^. The global prevalence of CI among community dwellers is over 15% and is affected by age, gender, education level and region of study sites^2,3^.

Recent studies have highlighted type 2 diabetes (T2D) as one of the primary causes of CI^4,5^. Experimental studies, in line with epidemiological findings, have demonstrated a cause-effect relationship between T2D and CI, T2D as a cause and CI as an effect. Both disorders have the same symptoms with insulin resistance (IR) as the most important^4,6^. IR leads to beta- amyloid (Aβ) and tau accumulation, a main diver of CI^7–9^. Researchers suggested that obesity can reinforce the correlation between T2D and CI by disrupting adipokines secretion and blood–brain barrier (BBB) function^4^.

After secreting by adipocytes, the adipokines adiponectin and leptin can crosse BBB and bind to their hippocampal receptors. When binds to their receptors, they can phosphorylate and activate AMP-activated protein kinase (AMPK)^10^. AMPK phosphorylates and activates peroxisome proliferator-activated receptor-gamma coactivator 1 (PGC-1α), which in turn increases the transcription of peroxisome proliferator-activated receptors (PPAR α), the major regulator of insulin and glucose metabolism. PPAR α can also inhibit beta-secretase 1 (BACE1) transcription, a speed-limiting factor in the synthesis of Aβ^11^. PGC-1 α could also inhibits BACE1 transcription^12^.

Binding leptin to its receptor can also trigger phosphoinositide 3-kinases (PI3K) pathway which finally increase the level of dephosphorylated glycogen synthetize kinase 3 beta (Dep-GSK3ß), a main regulator of hyperphosphorylated TAU accumulation. Dep-GSK3β levels could also increase through phosphorylating mitogen-activated protein kinase (MAPK) (i.e. the more phosphorylated MAPK the more dephosphorylated GSK3ß) by adiponectin. Furthermore, several studies^13,14^ showed a cross talk between MAPK and AMPK. Accordingly, circulating levels of adiponectin and leptin, their receptors levels in the hippocampus as well as the points of their interaction (i.e. GSK3ß and AMPK) should be the center of attention when studying T2D induced CI^15^.

Studies have shown that blood levels of adiponectin and leptin and their receptors are higher in female than male^16–19^. Several major reasons have been proposed for this difference including higher levels of body fat in the female^17^ and innate characteristics of female fat cells (i.e. serum leptin and adiponectin levels are higher in female despite the same body fat mass)^19,20^. Subcutaneous is more active than visceral fat in producing adipokines and the higher levels of subcutaneous fat could also explain women higher levels of leptin and adiponectin^16,19^. Furthermore, it has been reported that female infants have twice the concentration of leptin in their umbilical cord, suggesting that gender differences begin in utero^17,21,22^. Chen et al.^22^ reported that the number of X chromosomes affects adipose tissue function in rats. They added that there are several genes on the X chromosome which control obesity and metabolic disorders.

Exercise is a well-known, inexpensive, and safe treatment for metabolic disorders^23–26^. Researchers believe that a part of the beneficial effects of exercise on metabolic disorders is mediated by adiponectin and leptin^27–29^. Depending on the exercise type, volume, and intensity, leptin and adiponectin show different responses to exercise^20,30^. In addition, it has been shown that the effects of exercise training on serum leptin and adiponectin levels is sex dependent^31,32^. In our previous study, we showed that high intensity interval training (HIIT) could attenuate T2D included CI in male rats through adiponectin signaling^6^. It was also shown that leptin affects the same molecular pathways^33^. In this study, we aimed to compare the attenuating effect of 8-week HIIT on T2D induced CI between male and female rats with the special focus on adiponectin and leptin.

## Material and methods

### Animals

The study was performed with the approval of the Animal Care and Ethics Committee of Kerman University of Medical Sciences (KUMS) in accordance with the institutional guidelines of KUMS (Ethics Approval Code: IR.KMU.REC.1401.033) and in compliance with the ARRIVE guidelines. Then, we purchased 56 eight-week-old Wistar rats (males and females), with an average weight of 200–250 g, from the animal farm of KUMS and kept them at 23 ± 1.4 °C and a 12:12 dark–light cycle in special polycarbonate cages. All animals had free access to water and food. After being accustomed to the laboratory environment, the animals were randomly assigned to 4 groups (each group included 14 rats, 7 females and 7 males): control (Con), type 2 diabetes (T2D), exercise (EX), and type 2 diabetes + exercise (T2D + EX). The Ex and T2D + EX groups performed eight weeks of HIIT.

### Induction of type 2 diabetes

Animals in T2D and T2D + EX groups were fed a high-fat diet (HFD) for 2 months, while the animals in Con and EX groups were fed a normal and regular diet. The HFD was purchased from the Isfahan Royan Research Institute. HFD includes: 60% fat (245 g of lard and 25 g of soybean oil), 20% carbohydrate (125 g Lodex10 and 72.8 g sucrose), 19% protein (200 g of casein and 3 g of cysteine), 50 g of fiber (Solca Floc), 50 g of minerals, 3 g of vitamins, and 0.5 g of dye^6,34^ (Table 1). Rats’ weight and food intake were measured weekly. The criterion for obesity was a weight of 350 g^34^. After 2 months, the animals fasted for 12 h, and a single dose of 35 mg/kg of streptozotocin (STZ) was injected intraperitoneally. Animal blood glucose was measured 2 weeks after STZ injection using a glucometer. Animals with fasting blood glucose (FBG) levels above 300 mg/dl were considered diabetic and included in the study^35^. The fasting time before STZ injection and also before sampling at the end of the study was 12 h.

**Table 1:** High-fat and regular diet ingredients.

Diet ingredients	Fat	Carbohydrate	Protein	Fiber/Mineral/Vitamin	Total energy
Regular diet	10%	70%	19%	1%	341 Cal/100 g or 1432 J/100 g
High-fat diet	60%	20%	19%	1%	429 Cal/100 g or 1802 J/g

### Exercise protocol

Initially, in the familiarization phase, EX + T2D and EX groups ran on a treadmill twice a day for 5 days, 10 min per day with a zero-incline and a speed of 8 m per minute. Then an incremental running test was performed to determine the rats’ maximum speed (V_max_). In this test, the rats ran for 2 min at a speed of 6 m per minute, and every 2 min, 2 m per minute were added to the speed until they could not maintain this speed. The last attempt of each rat was considered Vmax. The main training protocol was performed for eight weeks five days a week. The exercise protocol is shown in Table 2. At the beginning and end of each session, the rats run on a treadmill for 5 min at an intensity of 40–50% of V_max_ for warm up and cool down. It should be noted that rats’ v_max_ was remeasured every two weeks and used for designing the training protocol at the next 2 weeks^6,36–39^.

**Table 2:** High-intensity interval training (HIIT) protocol.

week	slope	Frequency	intervals	High-intensity interval duration (min)	Low-intensity interval duration (min)	High-intensity interval velocity (Vmax)	Low-intensity interval velocity (Vmax)	Total exercise time in a session (min)
1	0	5	4	2	1	80	50	12
2	0	5	4	2	1	85	50	12
3	0	5	6	2	1	85	50	18
4	0	5	6	2	1	90	50	18
5	0	5	8	2	1	90	50	24
6	0	5	8	2	1	95	50	24
7	0	5	10	2	1	95	50	30
8	0	5	10	2	1	100	50	30

### Sampling

All animals were sacrificed 48 h after the last training session by intraperitoneal injection of ketamine 80 mg/kg and xylazine 10 mg/kg. Serum and hippocampal tissue were used bilaterally to study the variables^40^.

### Western blotting method

Western blotting was used to assessed the expression of AMPK, GSK3ß, Aβ, Tau, APNR1 & 2 and LEPR in rat hippocampus. First, samples were prepared. The target proteins separated on the gel and were transferred to nitrocellulose paper with pores of 0.45 μm at 0.5 amps for 1.5 h. For this purpose, first pour some buffer in a clean container, put the gel in the buffer for at least 10 min after cutting the compacting part. Then, with the help of pliers, we cut 1 piece of nitrocellulose paper and several filter pads exactly the same size as the gel and transferred the two sponges that were placed on the sides of the membrane and gel to the buffer until they were completely wet. Then we put the above components on top of each other, finally we fixed the blot sandwich in the relevant plastic frame and placed it in the blot tank which was filled with buffer to the appropriate height. The specific number of primary antibodies was (1:1000) and GAPDH was mixed and diluted, incubated for 16 to 18 h. The membrane is immersed in a secondary antibody solution of appropriate concentration in an immunoassay buffer for 1.5 h with shaking, twice rinsing with saline solution (TBST) and once with peripheral blood smear (PBS) was performed. The membrane was placed in a sufficient amount of chromogenic substrate (TMB) solution until the bands appeared. The reaction was stopped by adding distilled water and evaluated after the bands appeared^41–44^.

### ELISA

Serum leptin , insulin and adiponectin concentrations were measured by ELISA kits according to the manufacturer's instructions (Rat ELISA Kit, Eastbiopharm)^45,46^.

### Statistical analysis

The data are reported as mean ± standard deviation (SD). Statistical analysis was performed using GraphPad Prism version 8. Normality and homogeneity of variances were assessed using Shapiro–Wilk and Leven tests, respectively. Two-way ANOVA followed by Tukey's post-hoc were used to analyze the data. P values less than 0.05 was considered statistically significant^47,48^.

### Ethics approval

All stages of keeping and scarifying the animals were performed according to the rules of the ethics committee of Kerman University of Medical Sciences (Ethic code: IR.KMU.REC.1401.033).

## Results

We assessed FBG to confirm our diabetes induction method. Our results showed that blood glucose was significantly increased in both male and female rats after diabetes induction (2 months of HFD and STZ injection) (month 2) compared with the pretest (month 0) in T2D and T2D + EX group (P = 0.02), with no significant difference between these groups. In addition, HIIT reduced blood glucose significantly (P < 0.05). We saw no significant effect for gender (P = 0.09) (Table 3). Also, the animals body weight results was shown in Table 4.

**Table 3:** FBG (mean ± SD) before starting the intervention (month 0), after diabetes induction (2 months of high-fat diet and STZ injection) (month 2), and 48 h after the last training session (month 4) in all groups.

Gender	Group	Pre-test (Month 0)	Month 2	Post-test (Month 4)
Female	Con	140 ± 6	154 ± 7	165 ± 9
	T2D	147 ± 7	355 ± 4*	251 ± 13^#^
	EX	142 ± 8	155 ± 9	160 ± 11
	T2D + EX	150 ± 7	373 ± 2*	203 ± 11^#^
Male	Con	192 ± 8	198 ± 9	205 ± 12
	T2D	188 ± 9	378 ± 7*	272 ± 7^#^
	EX	190 ± 8	192 ± 8	191 ± 8
	T2D + EX	189 ± 7	396 ± 7*	212 ± 10^#^

*FBG* Fasting blood glucose, *Con* control, *T2D* Type 2 diabetic (STZ injected), *Ex* exercise only, and *T2D* + *Ex* Type 2 diabetic + exercise.

*Significant difference with pretest.

^#^Significant difference with month 2.

**Table 4:** Animals’ weight (mean ± SD) before starting the intervention (month 0), after diabetes induction (2 months of high-fat diet and STZ injection) (month 2), and 48 hours after the last training session (month 4) in all groups.

Gender	Group	Pre-test (Month 0)	Month 2	Post-test (Month 4)
Female	Con	219 ± 16	221 ± 14	218 ± 15
	T2D	220 ± 10	367 ± 23*	271 ± 18#
	EX	218 ± 14	219 ± 13	218 ± 15
	T2D + EX	217 ± 17	352 ± 21*	292 ± 18^#^
Male	Con	284 ± 18	285 ± 14	287 ± 13
	T2D	285 ± 12	398 ± 25*	307 ± 15^#^
	EX	271 ± 9	283 ± 11	294 ± 8
	T2D + EX	282 ± 10	381 ± 21*	319 ± 21^#^

*Con* control, *T2D* Type 2 diabetic, *Ex* exercise, and *T2D* + *Ex* Type 2 diabetic + exercise.

*Significant difference with pretest.

^#^Significant difference with month 2.

Animals’ weight significantly increased in T2D and T2D + Ex groups after diabetes induction (month 2) in both male and female rats. In addition, the weight was decreased in T2D and T2D + EX groups, with more decrease in the T2D group (P < 0.05) in post-test (month 4). The female rats have lower body weights in all groups (P < 0.05) (Table 3).

**Figure 1:**
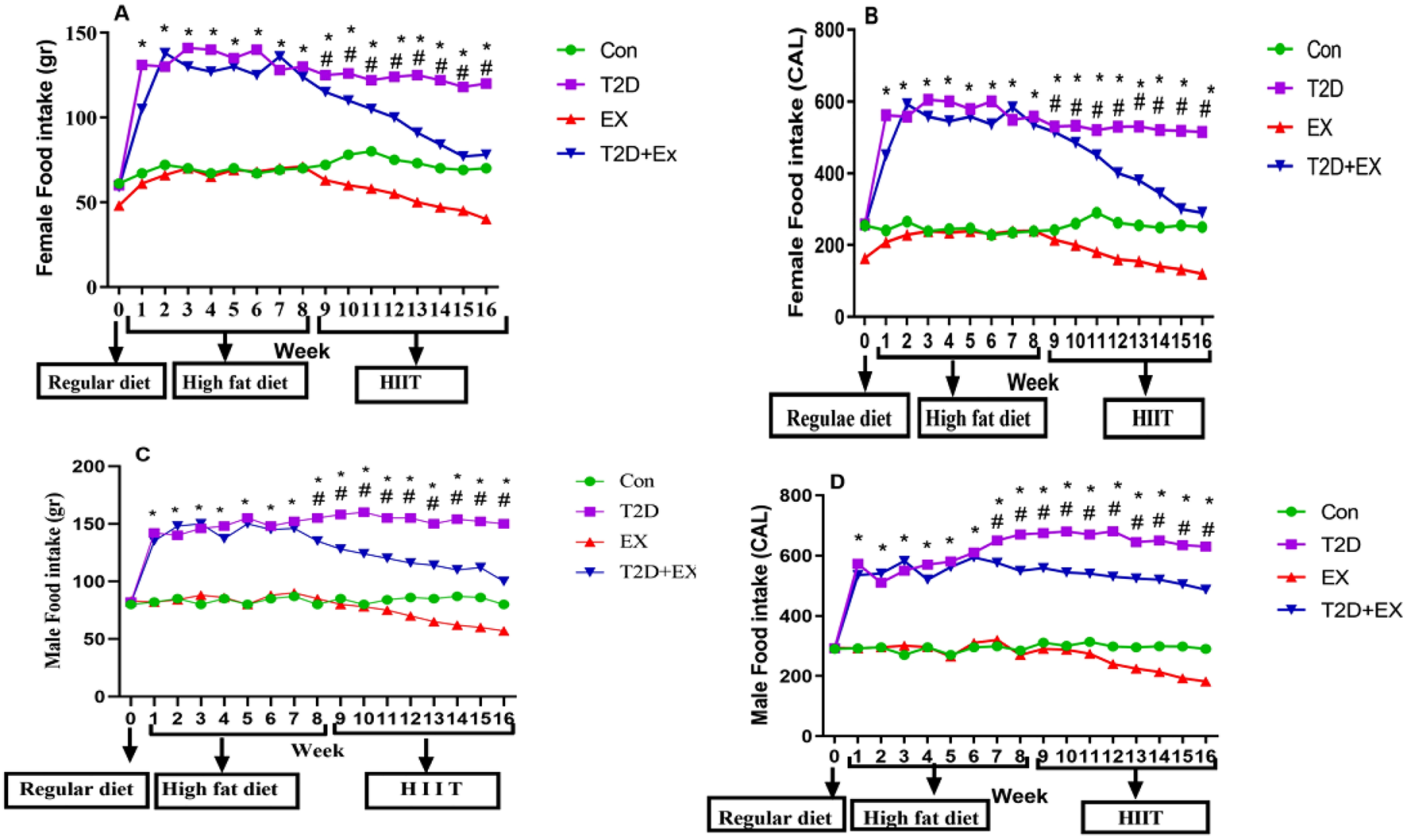
Food intake in calories ((**B**) and (**D**)) and in grams ((**A**) and (**C**)) in all groups (n = 7 in each group). We measured these parameters before the high-fat diet (month 0), after the high-fat diet and STZ injection (month 2), and 48 h after the 8-week HIIT program (month 4). *Con* control, *T2D* Type 2 diabetes, *Ex* exercise, and *T2D* + *Ex* Type 2 diabetes + exercise. ^**#**^Significant difference between T2D and T2D + EX (8–16 week), ^**&**^Significant difference between EX and T2D + EX (8–16 week), ^**$**^Significant difference between EX and Con (8–16 week).

In the first week (familiarization) there was no significant different between groups in food intake but after two months of high-fat diet, food intake in T2D and T2D + EX groups increased significantly in both sex (P < 0.0001) with no significant difference between them. After STZ injection, and started exercise training, rats’ food intake decreased significantly in EX and T2D + EX groups in both sex (P < 0.0001) but there was no sex difference. Finally, after 8-weeks HIIT rats’ food intake decreased significantly in EX and T2D + EX groups in both sex (P < 0.0001) without significant difference between sexes (Fig. 1A–D).

**Figure 2:**
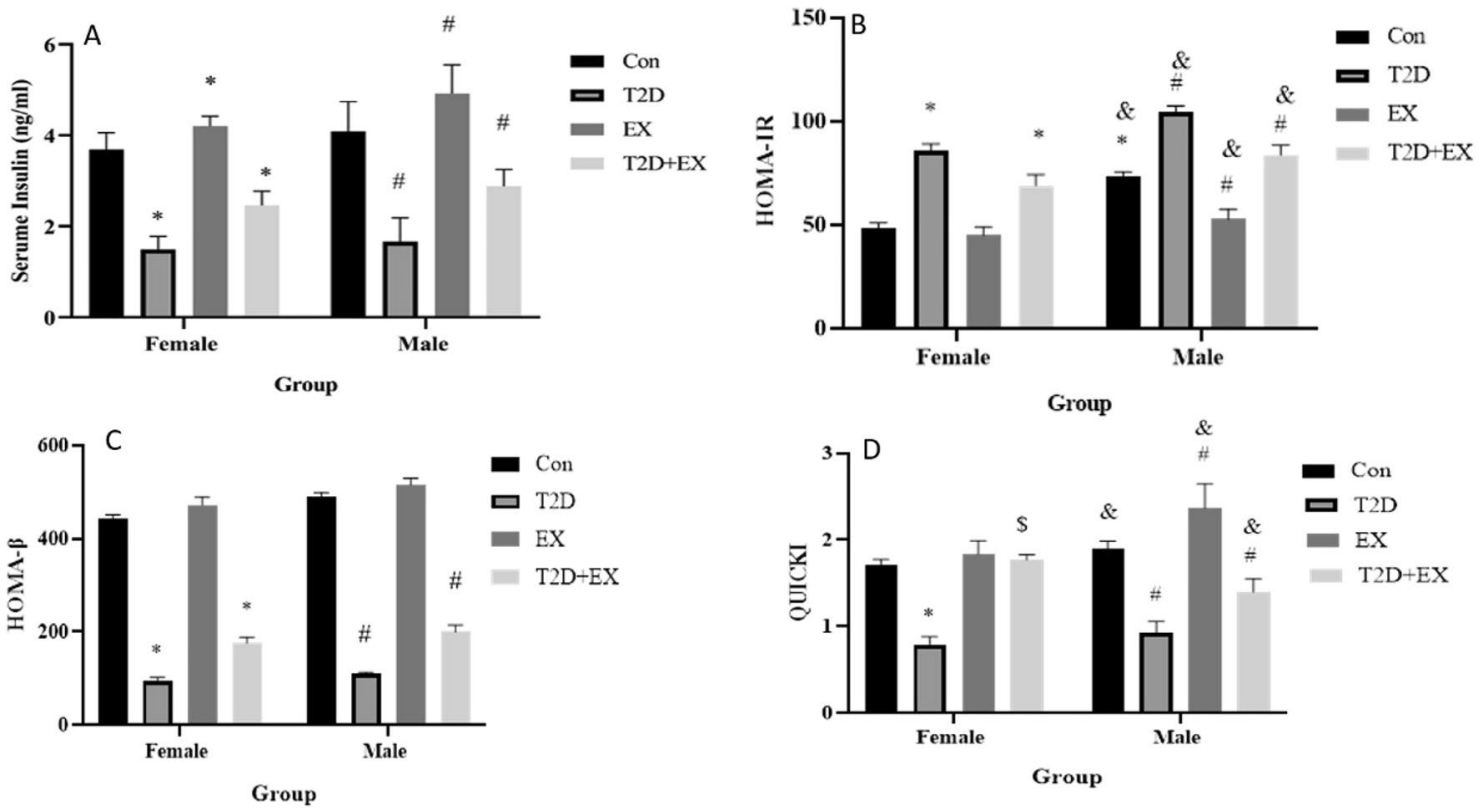
Serum insulin levels (**A**), HOMA-IR (**B**), HOMA-B (**C**) and Quicki (**D**) (mean ± SD) in all groups. *Significant difference with Con_female_, ^#^Significant difference with Con_male_. ^$^Significant difference with T2D + EX_female_. ^&^Significant difference with a female peer. *Con* control, *T2D* Type 2 diabetic, *Ex* exercise, and *T2D* + *Ex* Type 2 diabetic + exercise.

Our results showed the same result in both genders for serum insulin levels. Serum insulin level was decreased by T2D and increased by EX (*P*_*female*_ = 0.02, *P*_*male*_ = 0.02). In addition, a significant interaction was seen between T2D and EX (*P*_*female*_ = 0.02,* P*_*male*_ = 0.02). The interaction between gender and T2D, gender and EX, and gender, T2D and EX were insignificant (*P* > 0.05) (Fig. 2). To confirm these data, we assessed the index of pancreatic Beta cell function (i.e., HOMAβ), which showed the same results in both genders with no significant effect for gender (P > 0.05) except that Ex did not have a significant difference with Con. The interactions were also insignificant (P > 0.05) (Fig. 2). In addition, we checked the effect of T2D and EX on IR (i.e. HOMA-IR), and insulin sensitivity (i.e. QUICKI) indices. In female rats, while T2D increases and decrease HOMA-IR and QUICKI (*P* < 0.05), respectively, EX failed to change these indices significantly (*P* > 0.05). However, there was a significant interaction between T2D and EX in both indices (P < 0.05). The same result was seen in male rats except for significant effects of EX. EX decreases HOMA-IR and increases QUICKI in male rats (*P* < 0.05). A gender effect was seen for HOMA-IR, with a higher level in T2D female rats. Furthermore, the interaction between T2D, EX, and gender was significant with lower levels in the male rats (P < 0.05). The gender difference was seen for QUICKI in Con, EX and EX + T2D groups (P < 0.05) (Fig. 2).

To study if T2D and EX could affect LEP and APN levels, we assessed their levels in serum. APN levels in serum decreased in T2D and increased in EX groups compared to the Con group. In addition, T2D + EX showed higher levels of APN compared to T2D in both genders (P < 0.05). We also saw gender effects, with higher levels in females (P < 0.05). In addition, the female group of T2D + EX showed higher levels of APN than the same group of male rats (P < 0.05). This difference may reflect the different levels of basal APN rather than the different effects of HIIT.

**Figure 3:**
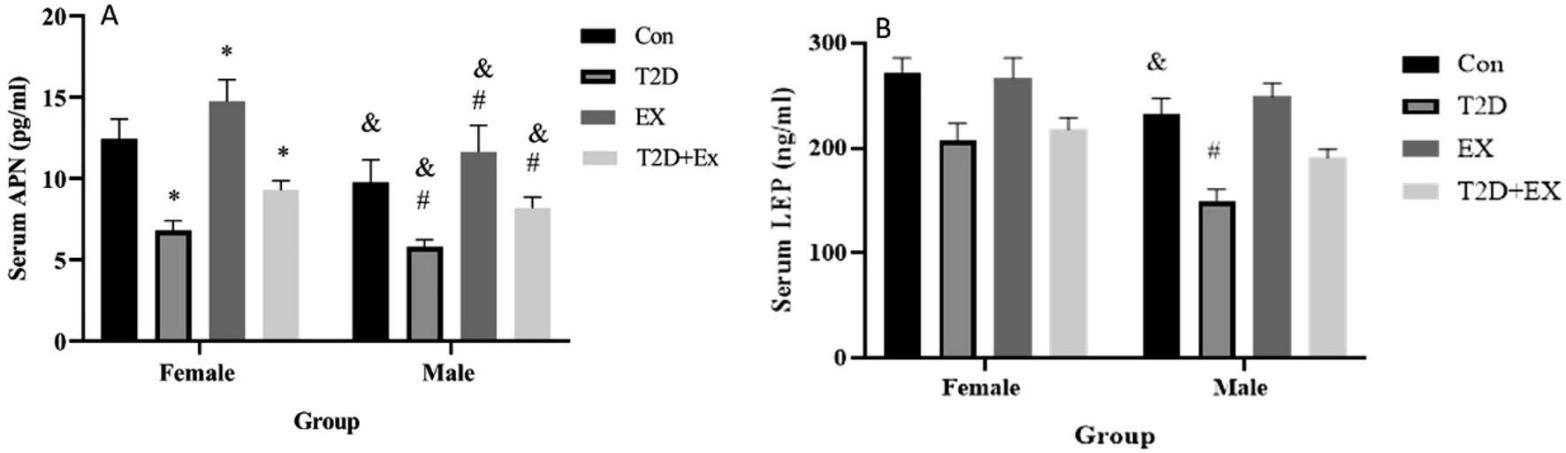
Serum level of APN (**A**) and Lep (**B**). (mean ± SD) in all groups. *Significant difference with Con female. ^#^Significant difference with Con male. ^&^Significant difference with a female peer. *Con* control, *T2D* Type 2 diabetic, *Ex* exercise, and *T2D* + *Ex* Type 2 diabetic + exercise.

In contrast with APN, LEP did not change in EX compared with Con. This was observed in both genders. The EX, T2D interaction was also insignificant. Nevertheless, a significant gender effect was seen and the LEP level was higher in females than males (P < 0.05). The interaction between gender, T2D, and EX was not significant (Fig. 3).

**Figure 4:**
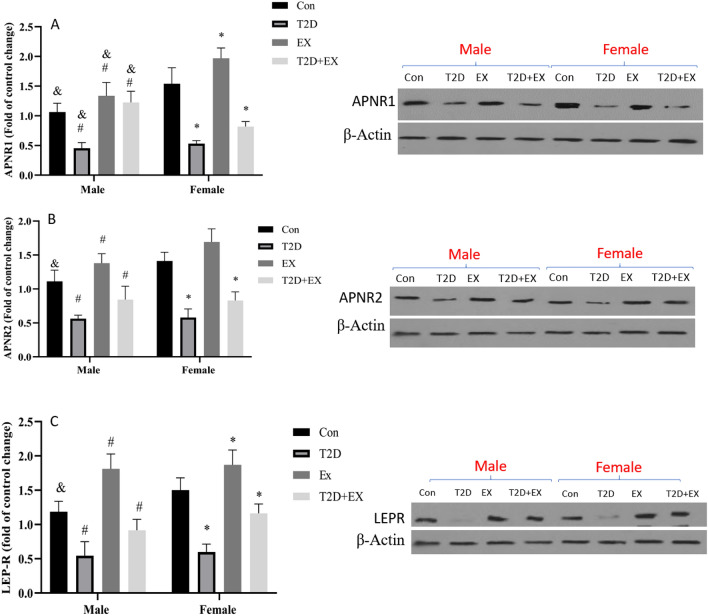
APNR1 (**A**), APNR2 (**B**) and LepR (**C**). (mean ± SD) in all groups.* Significant difference with Con female. ^#^Significant difference with Con male. ^&^Significant difference with a female peer. *Con* control, *T2D* Type 2 diabetic, *Ex* exercise, and *T2D* + *Ex* Type 2 diabetic + exercise. Original blots/gels are presented in Supplementary Fig. 1 which were cropped from different gels.

We measured the LEP-R and APN-R1, and APN-R2 levels in the hippocampus. Our results showed a similar pattern for all three receptors in both genders. LEP-R, APNR1, and APNR2 showed a significant reduction in T2D compared to Con (P < 0.05). However, EX could increase them significantly (P < 0.05). A significant interaction between T2D and EX has been seen for all three receptors confirming the positive effect of HIIT in diabetic rats (P < 0.05). A significant effect of gender was seen for all three receptors in the Con group, with higher levels in female rats (P < 0.05). In addition, a higher level of APNR1 was seen in the male T2D + EX group compared to its female peer (P < 0.05). These results highlight the vital role of APN1 (Fig. 4).

**Figure 5:**
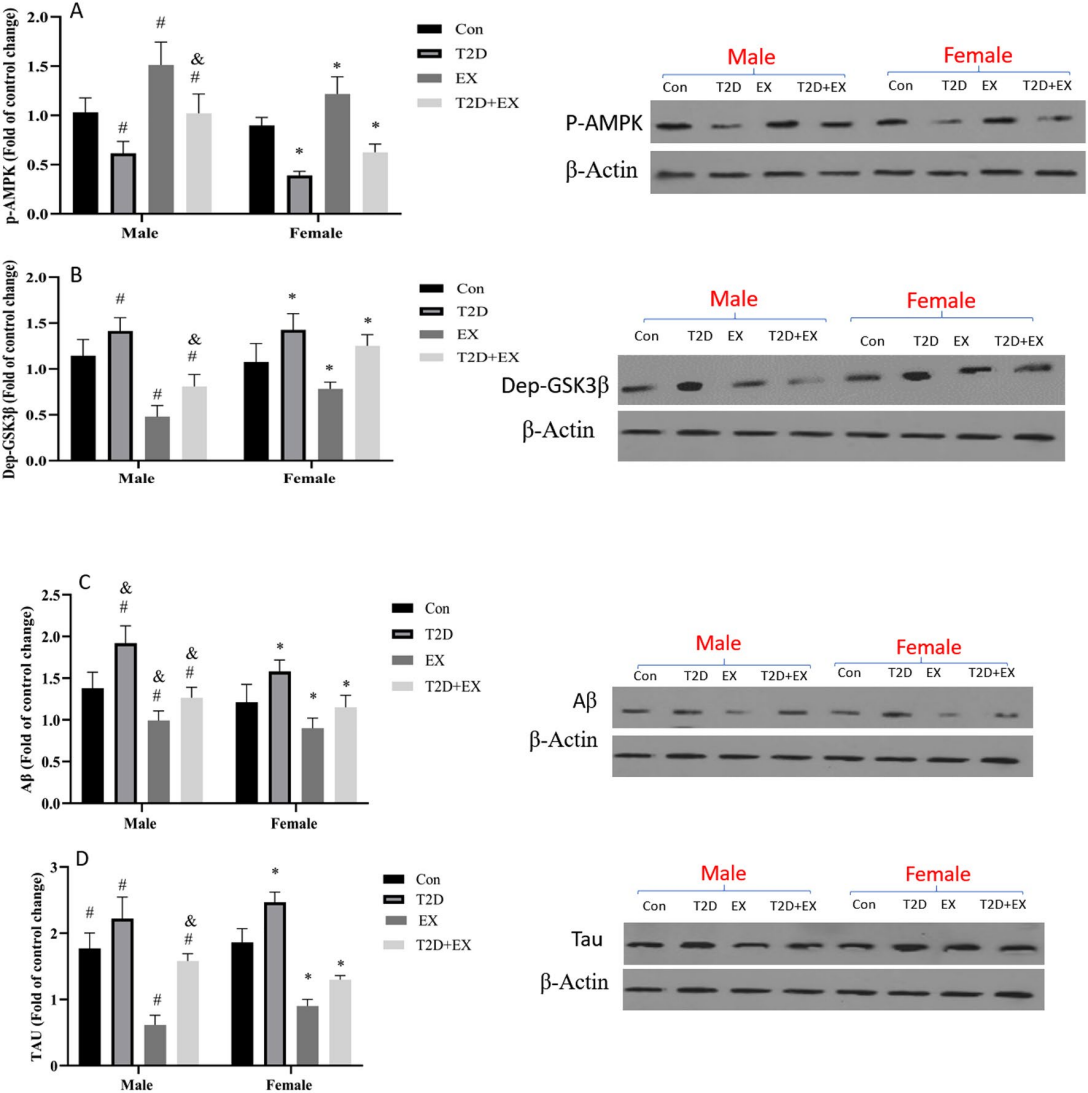
P-AMPK (**A**), Dep-GSK3B (**B**), AB (**C**) and Tau (**D**). (mean ± SD) in all groups. *Significant difference with Con female. ^#^Significant difference with Con male. ^&^Significant difference with a female peer. *Con* control, *T2D* Type 2 diabetic, *Ex* exercise, and *T2D* + *Ex* Type 2 diabetic + exercise. Original blots/gels are presented in Supplementary Fig. 1 which were cropped from different gels.

AMPK and GSK are the points where the regulatory hippocampal cascades of APN and LEP can interact to determine the net effect. Our results showed that the hippocampal P-AMPK (active form) decreased with T2D and increased with EX significantly in both genders (P < 0.05). Compared with females, diabetic male rats had a significantly higher level of AMP-K after the training period (P < 0.05). Dep-GSK (which stimulates TAU hyperphosphorylation) increased by T2D and decreased after 8-week EX in both genders. A significant interaction was seen for T2D and EX in both genders, which confirms the positive effects of HIIT in female diabetic rats (P < 0.05). The gender, T2D and EX interaction was significant, with the lower levels in female rats in T2D + EX group (P < 0.05) (Fig. 5).

βA and Tau, the main indices of cognitive impairments, showed the same results. Both of them increased in T2D rats and increased in EX rats (P < 0.05). A significant interaction between T2D and EX was also seen. The interaction between gender, T2D and EX were significant which showed a higher effect of HIIT in the male T2D + EX group (P < 0.05) (Fig. 5).

## Discussion

This study was designed to compare the attenuating effects of HIIT on APN and LEP signaling pathways in the hippocampus of male and female rats with T2D.

Our findings showed that T2D had destructive effects on all signaling molecules while HIIT could reverse these changes partly. Our findings revealed that APNR1, AMPK, GSK, TAU and Aβ expression were different between male and female rats after 8 weeks of HIITT. In addition, diabetes-induced IR was higher in male compared to female rats. While diabetes was induced in both gender the lack of a protective effect of estrogen may contribute to higher IR in male rats^49,50^. Added to this is the association between APN and IR in both genders. APN could reduce IR by decreasing triglyceride content in muscle and liver in obese and diabetic animals. This effect results from increased expression of molecules involved in fatty-acid metabolism such as PPAR-α^51,52^. Circulating APN was higher in female than male rats. The biological basis for this sex difference has been described in introduction. The sex difference in adiponectin could also be explained by an inhibitory effect of testosterone. Treatment of both sham-operated and castrated male mice with testosterone was accompanied by a reduction in serum APN^53^. In addition, our results showed that while APNR1 expression was higher in Con_female_ but this was reversed after 8 weeks of HIIT. Kaminska et al.^54^ reported that the expression of APNR1 is sex dependent and it is higher in male than female animals which is in consistent with our results. Animal breed and age may explain inconsistent results. It has been shown that the level of APNR1 decreased in male rats following T2D^55^. Furthermore, The stimulatory effect of exercise on APNR1 showed a dose dependent pattern and HIIT is considered as the most power full stimulant^56^. This stimulatory effect is shown to be mediated by serum testosterone in T2D rats^57^. This could be the reason for higher APNR1 expression in diabetic male than female rats after 8 weeks of HIIT. APNR1 inhibits Dep-GSK3β expression and due to higher level of APNR1 in male than female rats after HIIT, Dep-GSK3β expression showed the opposite pattern which is in line with our previous study^6^. It has been shown that APNR1 suppression exacerbates AD-like pathologies (i.e. increased Tau accumulation). One study showed that in ovariectomized female animals, the expression of APNR1 decreased and hippocampal accumulation of Tau increased compared to the healthy animals. In line with this result, our data showed lower accumulation of Tau in hippocampus of diabetic male compare to female rats after HIIT. APNR1 can also stimulate AMPK expression and this could be the reason for higher AMPK expression in male than female rats after HIIT. Brown et al. showed that AMPK expression was higher in male compared to female mice in response to 12 weeks of Endurance exercise^58^. On other hand, estrogen can increase AMPK expression in brain^59,60^ and this can explain the high AMPK in healthy, not exercised female than male rats.

In general, previous studies have described the importance of some types of exercise in regulated adiponectin pathway in peripheric tissues as well as in the brain. For example, it has been shown that HIIT induced PGC-1 and APNR1 gene expressions and improved insulin sensitivity in obese individuals^61^. Also, resistance training improved insulin signaling in skeletal muscle of T2D rats^62^. Cho et al. revealed that exercise training improves whole body insulin resistance via APNR1^63^. Furthermore, some researches showed that acute physical exercise increases adiponectin signaling in the hypothalamus of lean mice^64,65^.

Another important factor in cognitive impairment induced by T2D is accumulation of Aβ which is under direct control of AMPK and therefore decreased more in male than female rats after HIIT. To the best of our knowledge, this is the first study to compare sex difference in response to cognitive improvements induced by exercise training in T2D rats. However, some studies showed that exercise can reduced Aβ deposition in hippocampus^66–69^. It should be noted that testosterone can increase Aβ clearance^70^ and as exercise is an increase testosterone stimulator^71,72^, some studies considered testosterone a mediator of exercise induced Aβ clearance^32^.

## Conclusion

**Figure 6:**
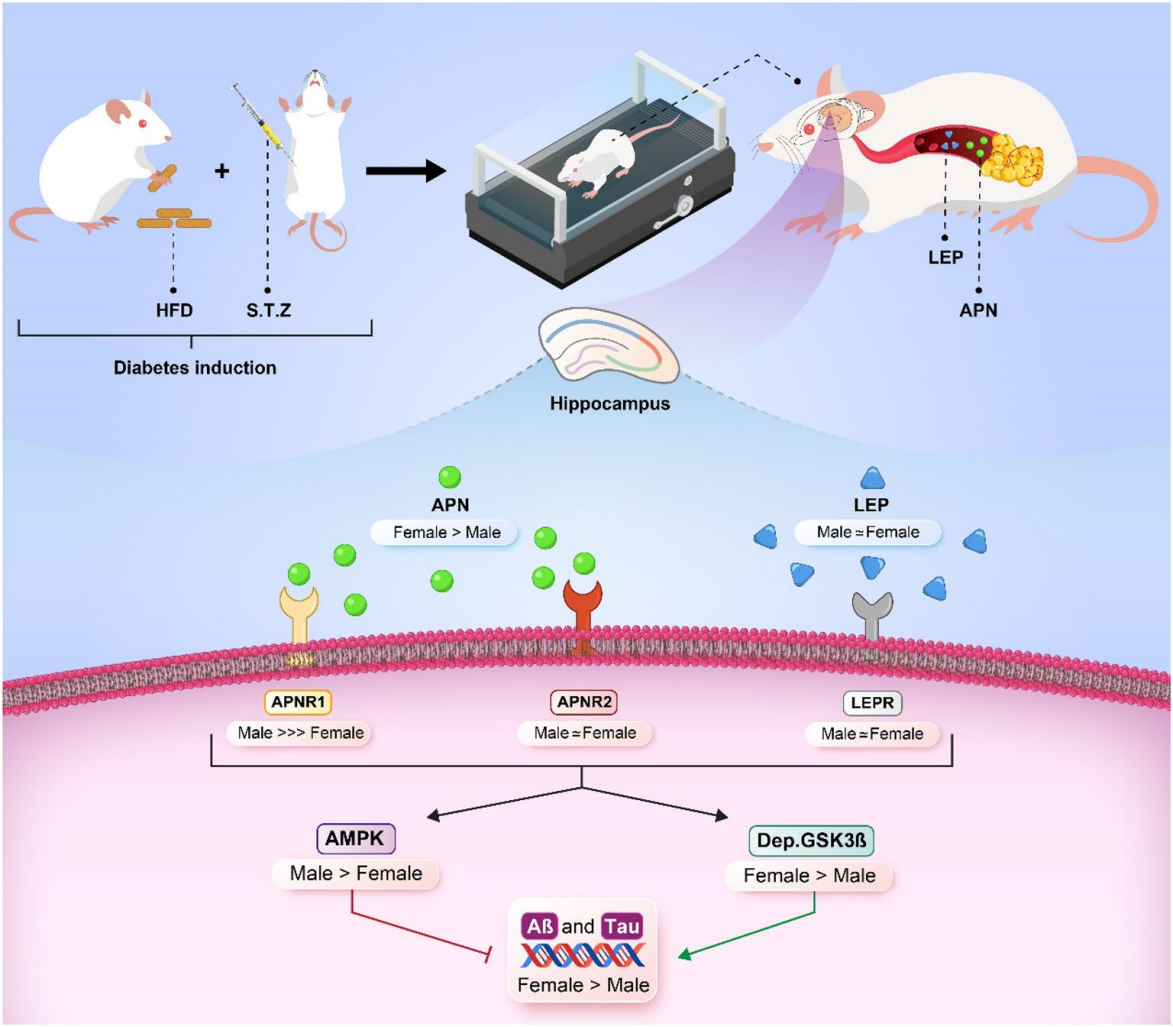
Adiponectin receptor 1 could explain the sex differences in molecular basis of cognitive improvements induced by exercise training in type 2 diabetic rats (HFD: High fat diet, LEP: Leptin, APN: Adiponectin).

In summary, we concluded that the risk of CI following T2D was more in female than male rats due to high deposition of Tau and Aβ in hippocampus but HIIT had more ameliorating effect in male animals than female. We believed that the difference in APNR1 expression and its downstream may be a main mechanism of this sex difference (Fig. 6).

### Supplementary Information

Supplementary Figures.

## Data Availability

The original contributions presented in the study are included in the article/supplementary material, and further inquiries can be directed to the corresponding author.
