# Supplementary material for: Adiponectin receptor 1 could explain the sex differences in molecular basis of cognitive improvements induced by exercise training in type 2 diabetic rats
Source: Sci Rep. 2023 Sep 27;13:16267. doi: 10.1038/s41598-023-43519-7 (PMC10533546; doi:10.1038/s41598-023-43519-7)
Supplement: Supplementary file 1 — Supplementary Figures. [file 41598_2023_43519_MOESM1_ESM.docx]

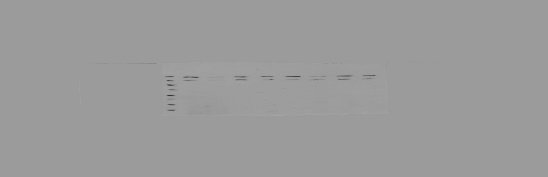

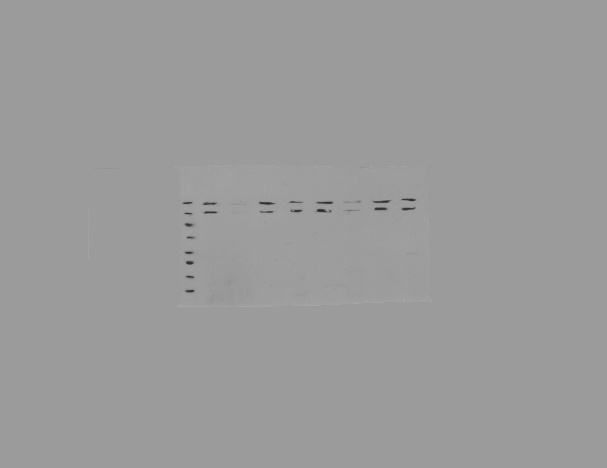

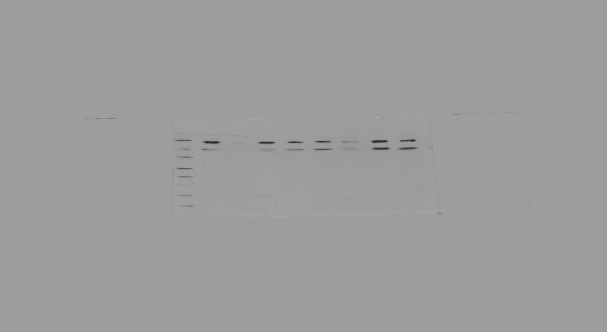

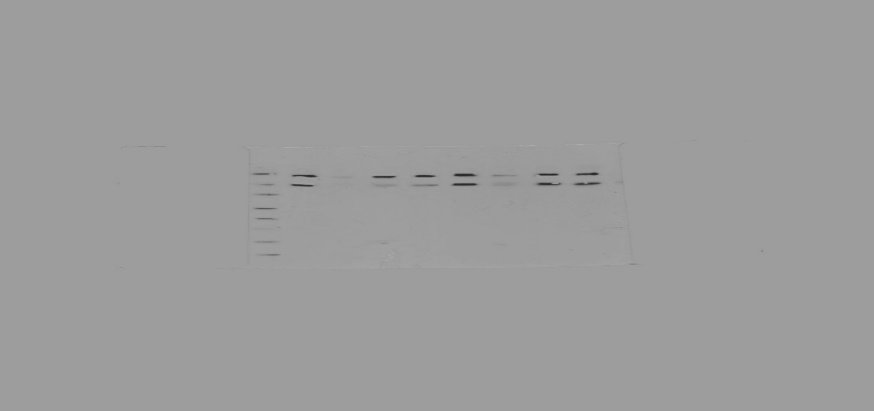

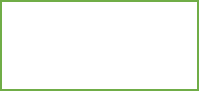

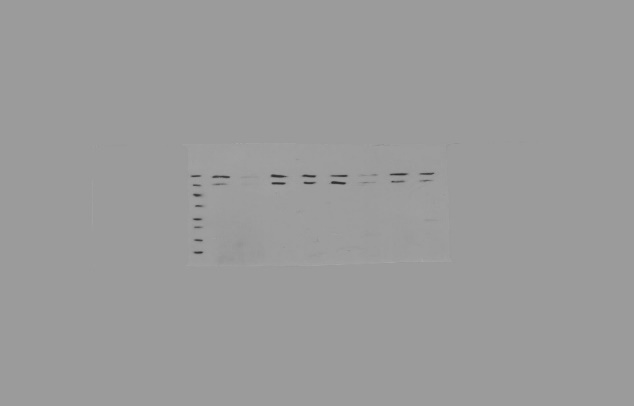

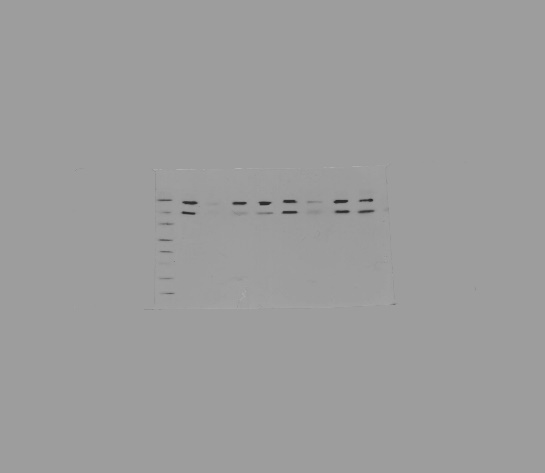

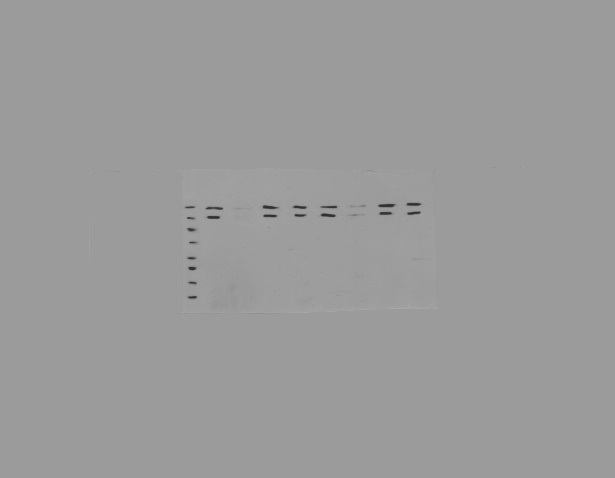


LEPR

LEPR

LEPR

LEPR

LEPR

LEPR

Female sample

Figure S1. Original immunoblots images of **Leptin receptor (LEPR)** in **males and females for Figure 4**. Note. all blot membranes were cut into stripes prior to hybridization with antibodies. Membrane edges are not clear in some cases, due to high signal-to-noise ratio of luminescence intensity.

CTL Db Ex Db/Ex

CTL Db Ex Db/Ex

Male sample

CTL Db Ex Db/Ex

CTL Db Ex Db/Ex

Male sample

Female sample

CTL Db Ex Db/Ex

CTL Db Ex Db/Ex

Male sample

Female sample

Male sample

LEPR

100 KDa

CTL Db Ex Db/Ex

CTL Db Ex Db/Ex

Male sample

Female sample

CTL Db Ex Db/Ex

CTL Db Ex Db/Ex

Male sample

Female sample

CTL Db Ex Db/Ex

CTL Db Ex Db/Ex

Female sample

Female sample

CTL Db Ex Db/Ex

Male sample

CTL Db Ex Db/Ex

Selected sample for manuscript


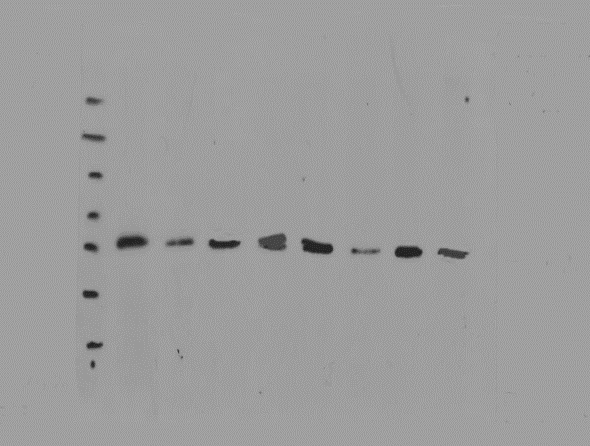

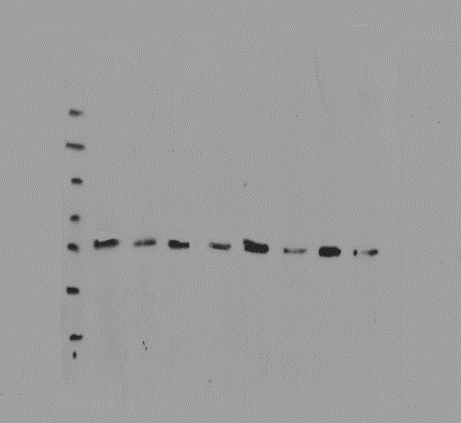

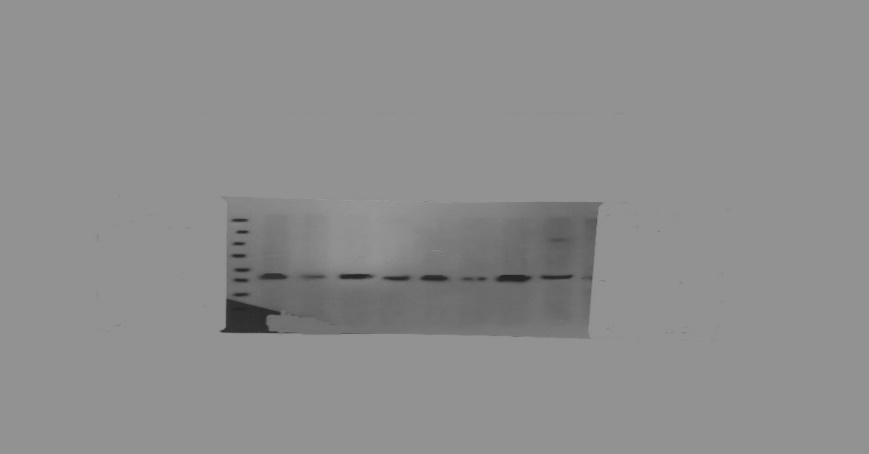

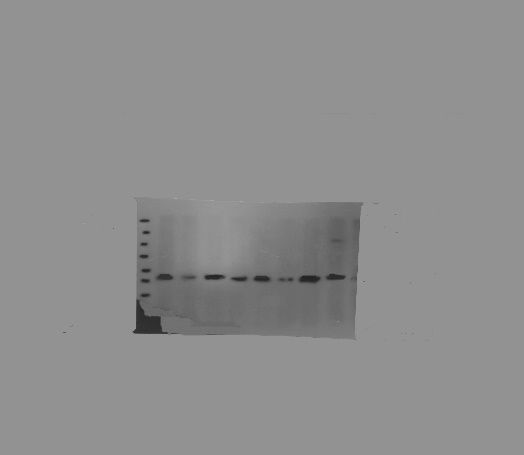

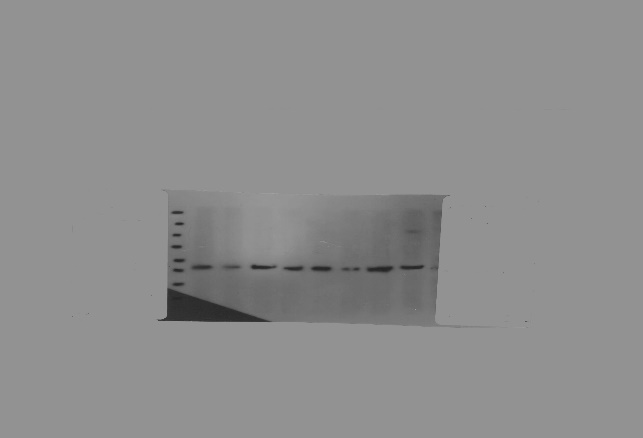

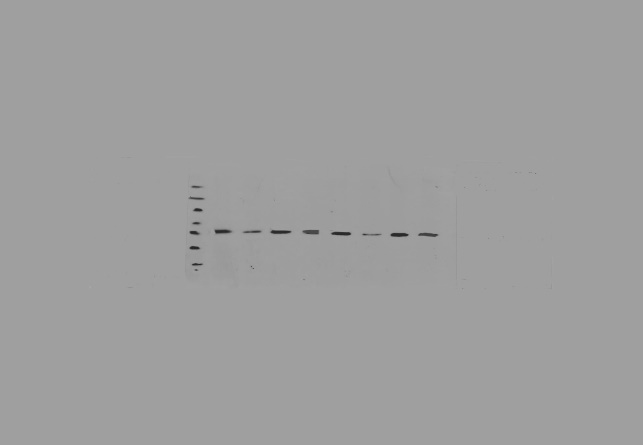

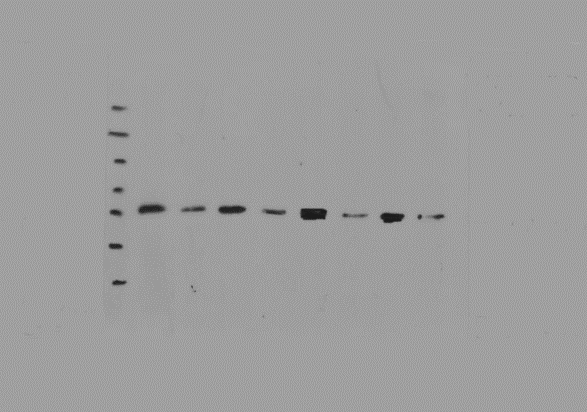


APNR1

APNR1

APNR1

APNR1

APNR1

CTL Db Ex Db/Ex

CTL Db Ex Db/Ex

Female sample

Male sample

CTL Db Ex Db/Ex

CTL Db Ex Db/Ex

Female sample

Male sample

CTL Db Ex Db/Ex

CTL Db Ex Db/Ex

Male sample

Female sample

Female sample

CTL Db Ex Db/Ex

CTL Db Ex Db/Ex

Male sample

Male sample

CTL Db Ex Db/Ex

CTL Db Ex Db/Ex

CTL Db Ex Db/Ex

CTL Db Ex Db/Ex

Female sample

APNR1

42 KDa

Selected sample for manuscript

Male sample

APNR1

Female sample

Figure S2. Original immunoblots images of **Adiponectin Receptor 1 (APNR1) in males and females for Figure 4**. Note. all blot membranes were cut into stripes prior to hybridization with antibodies. Membrane edges are not clear in some cases, due to high signal-to-noise ratio of luminescence intensity.

Male sample

Female sample

CTL Db Ex Db/Ex

CTL Db Ex Db/Ex


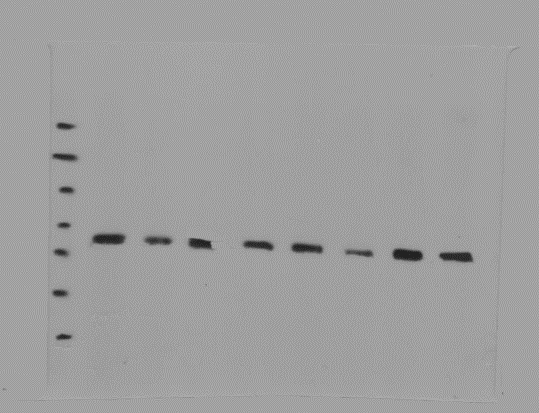

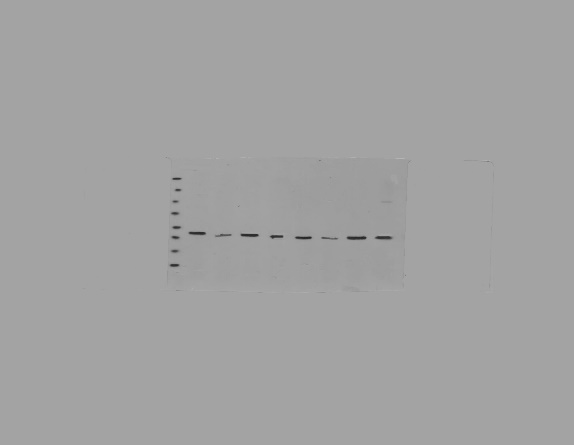

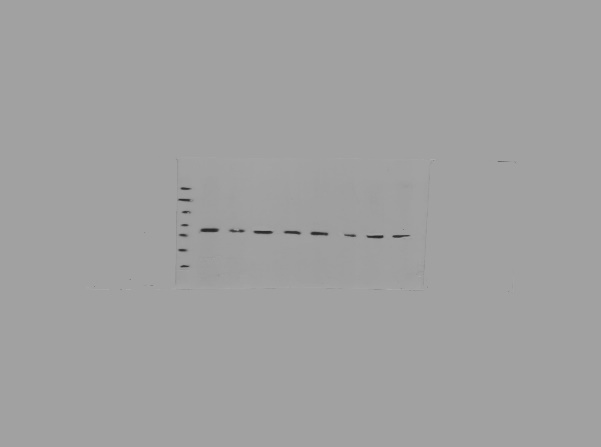

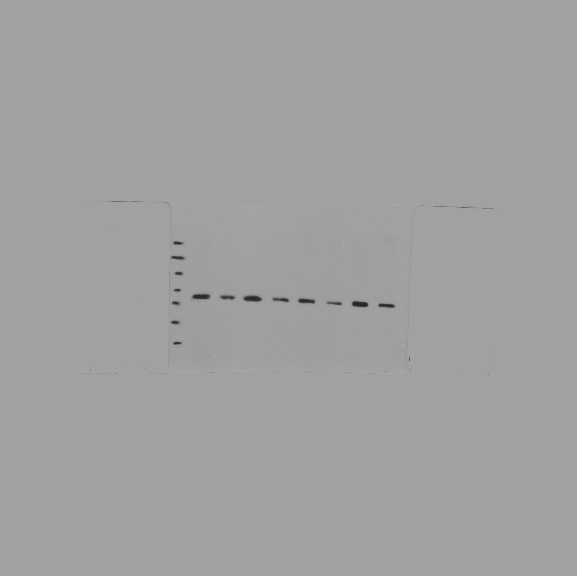

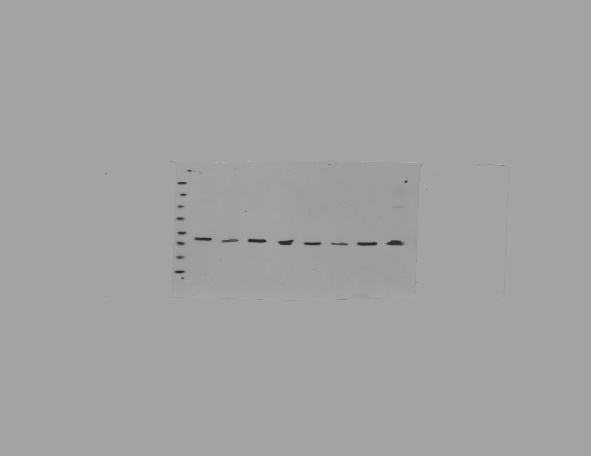

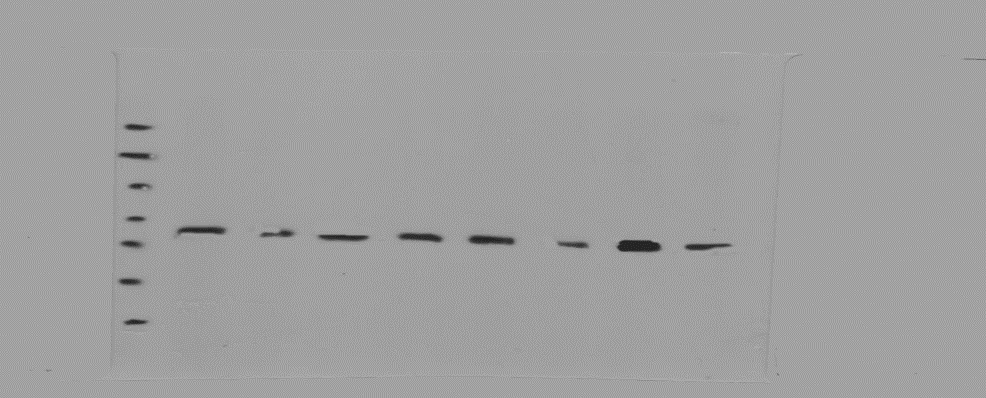

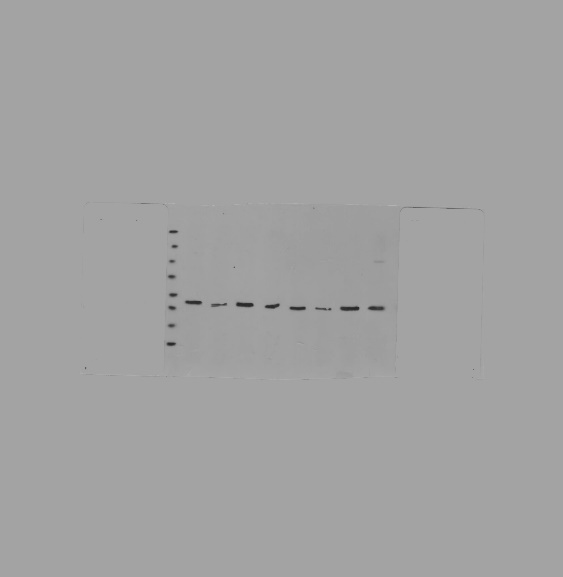


Male sample

CTL Db Ex Db/Ex

CTL Db Ex Db/Ex

Figure S3. Original immunoblots images of **Adiponectin Receptor 2 (APNR2) in males and females for Figure 4**. Note. all blot membranes were cut into stripes prior to hybridization with antibodies. Membrane edges are not clear in some cases, due to high signal-to-noise ratio of luminescence intensity.

APNR2

APNR2

APNR2

APNR2

APNR2

APNR2

CTL Db Ex Db/Ex

CTL Db Ex Db/Ex

Male sample

Female sample

Female sample

CTL Db Ex Db/Ex

CTL Db Ex Db/Ex

Male sample

Male sample

CTL Db Ex Db/Ex

CTL Db Ex Db/Ex

Female sample

Female sample

CTL Db Ex Db/Ex

CTL Db Ex Db/Ex

Male sample

Male sample

CTL Db Ex Db/Ex

CTL Db Ex Db/Ex

Female sample

APNR2

44 KDa

Selected sample for manuscript

Female sample

Male sample

Female sample


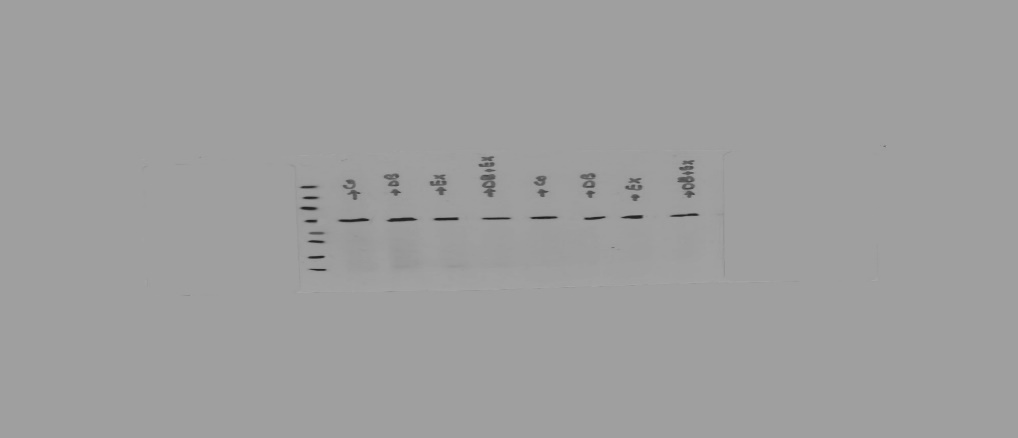

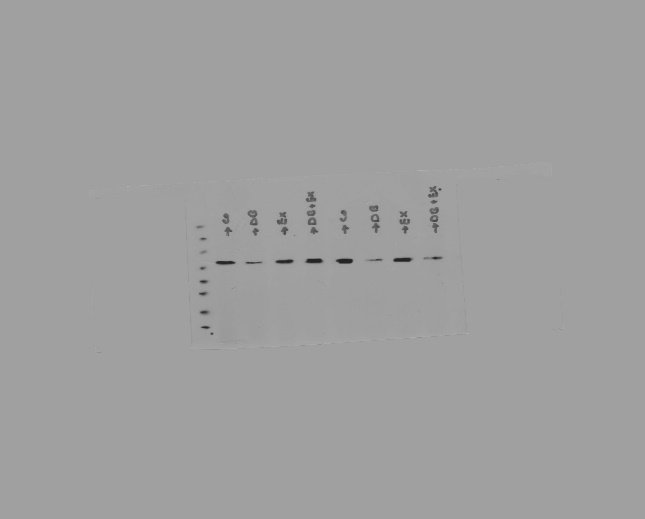

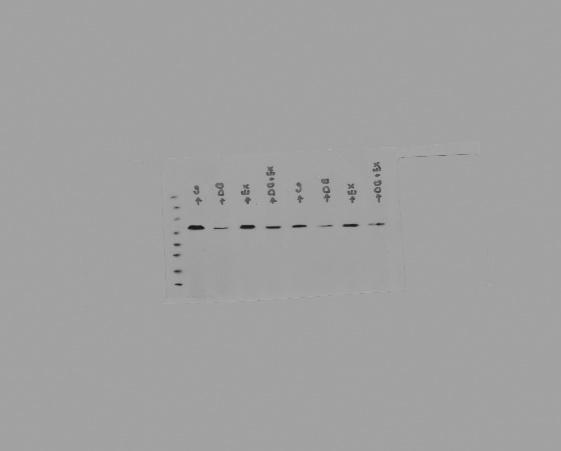

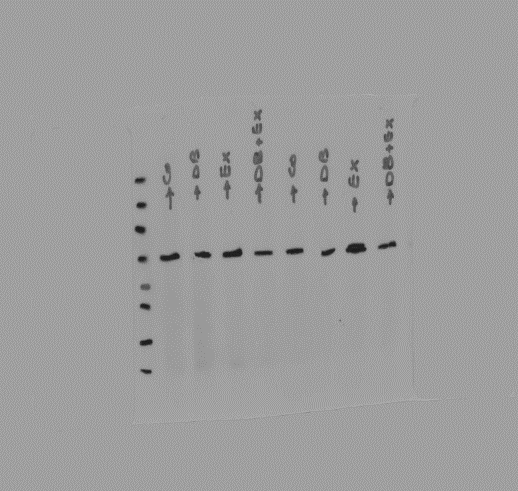

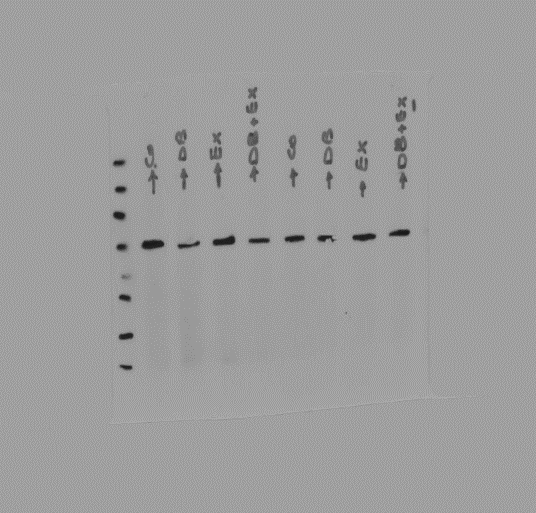

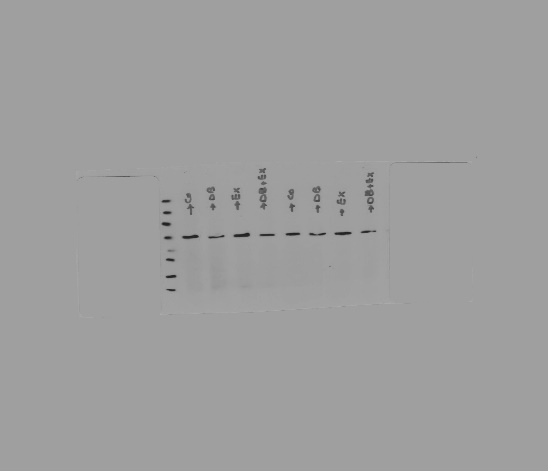

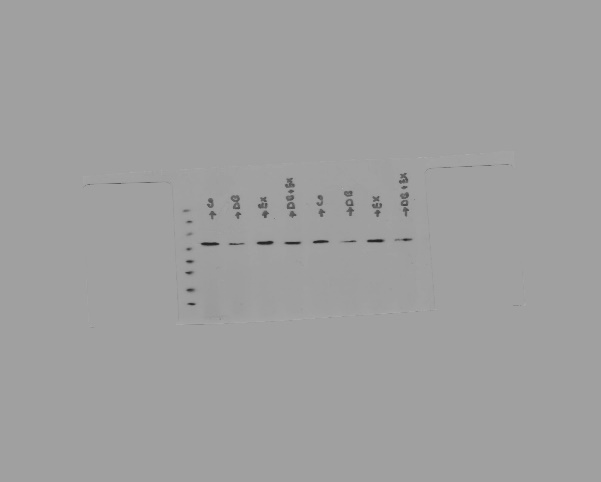


Figure S4. Original immunoblots images of **AMPK in males and females for Figure 5**. Note. all blot membranes were cut into stripes prior to hybridization with antibodies. Membrane edges are not clear in some cases, due to high signal-to-noise ratio of luminescence intensity.

CTL Db Ex Db/Ex

CTL Db Ex Db/Ex

AMPK

Male sample

Female sample

CTL Db Ex Db/Ex

CTL Db Ex Db/Ex

Female sample

Male sample

AMPK

AMPK

AMPK

CTL Db Ex Db/Ex

CTL Db Ex Db/Ex

Female sample

Male sample

Male sample

CTL Db Ex Db/Ex

CTL Db Ex Db/Ex

Female sample

AMPK

AMPK

Male sample

CTL Db Ex Db/Ex

CTL Db Ex Db/Ex

Female sample

Female sample

CTL Db Ex Db/Ex

CTL Db Ex Db/Ex

Male sample

CTL Db Ex Db/Ex

CTL Db Ex Db/Ex

AMPK

60 KDa

Selected sample for manuscript

Female sample

Male sample

Male sample


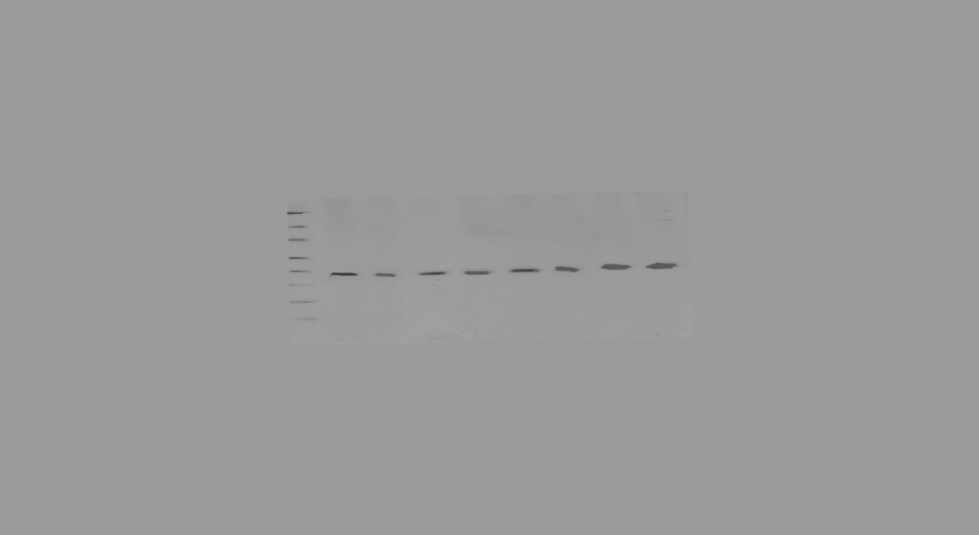

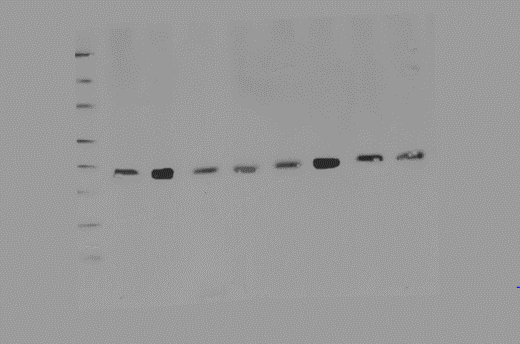

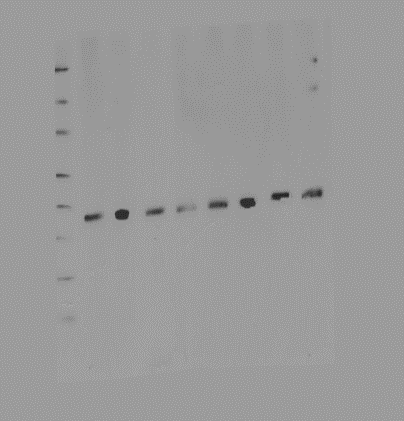

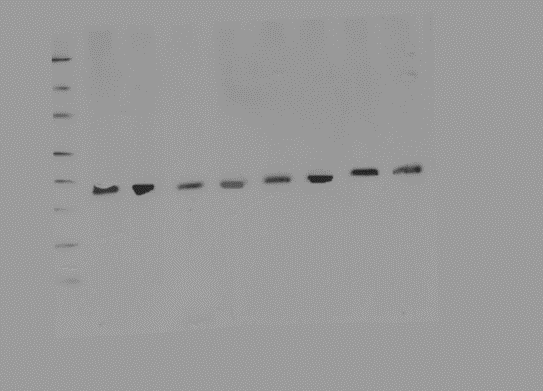

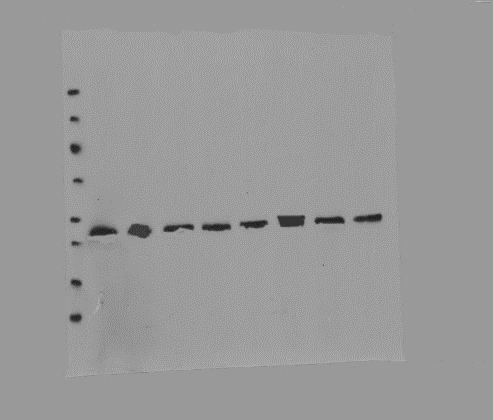

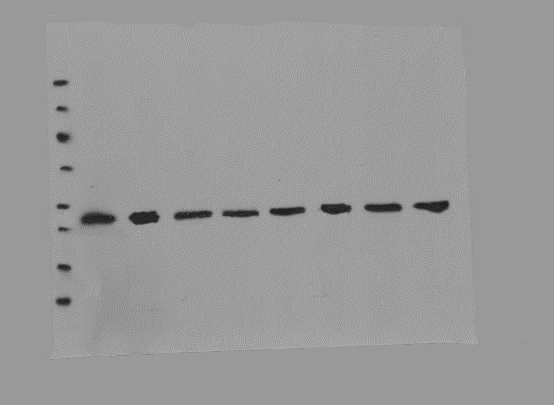

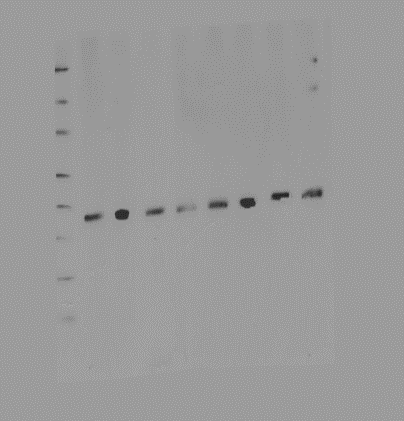


Male sample

Dep-GSK3

Figure S5. Original immunoblots images of **Dep-GSK3 in males and females for Figure 5**. Note. all blot membranes were cut into stripes prior to hybridization with antibodies. Membrane edges are not clear in some cases, due to high signal-to-noise ratio of luminescence intensity.

CTL Db Ex Db/Ex

CTL Db Ex Db/Ex

Dep-GSK3

Dep-GSK3

Male sample

Female sample

Male sample

Female sample

CTL Db Ex Db/Ex

CTL Db Ex Db/Ex

CTL Db Ex Db/Ex

CTL Db Ex Db/Ex

Dep-GSK3

CTL Db Ex Db/Ex

CTL Db Ex Db/Ex

Male sample

Female sample

Dep-GSK3

CTL Db Ex Db/Ex

CTL Db Ex Db/Ex

Female sample

Male sample

Dep-GSK3

CTL Db Ex Db/Ex

CTL Db Ex Db/Ex

Female sample

Male sample

Dep-GSK3

47 KDa

CTL Db Ex Db/Ex

CTL Db Ex Db/Ex

Selected sample for manuscript

Male sample

Female sample

Female sample


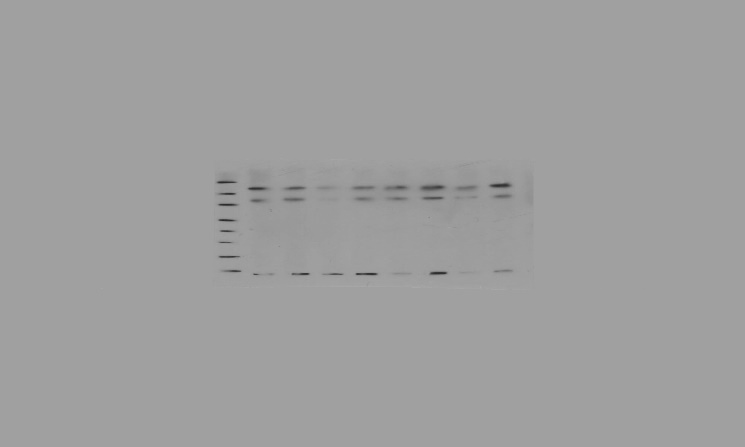

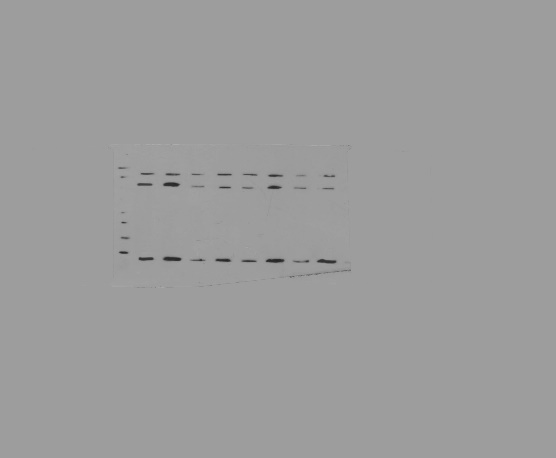

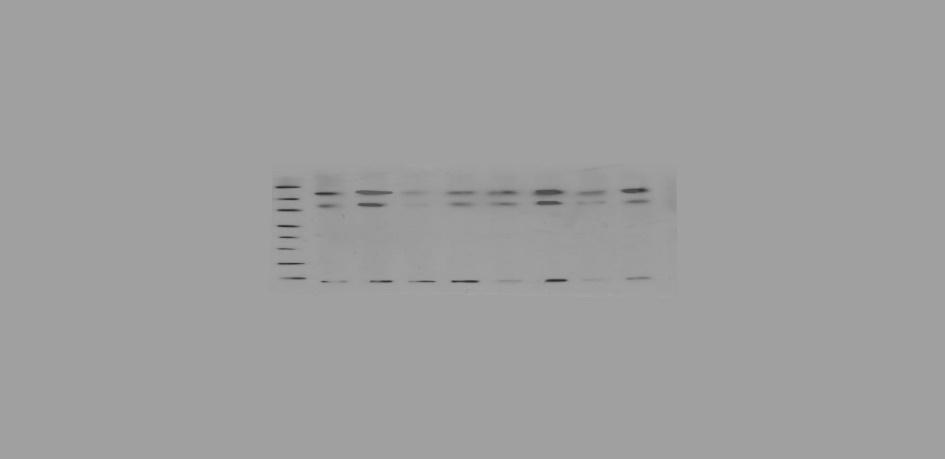

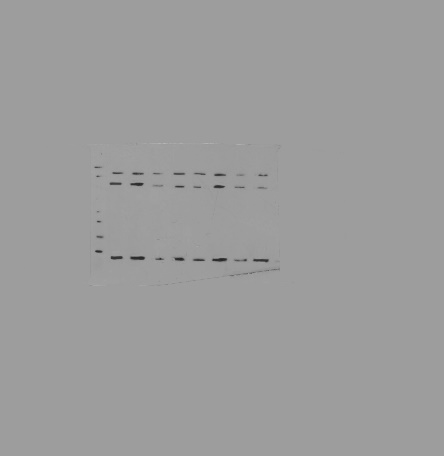

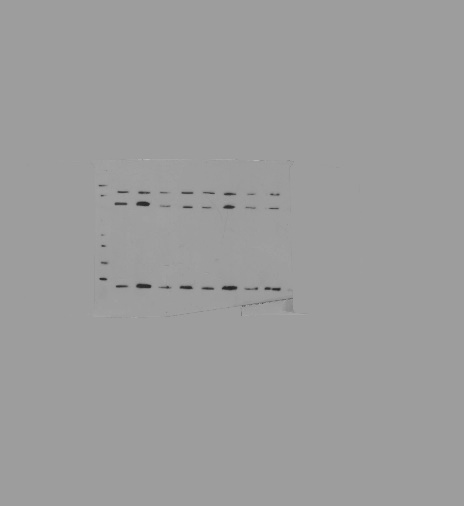

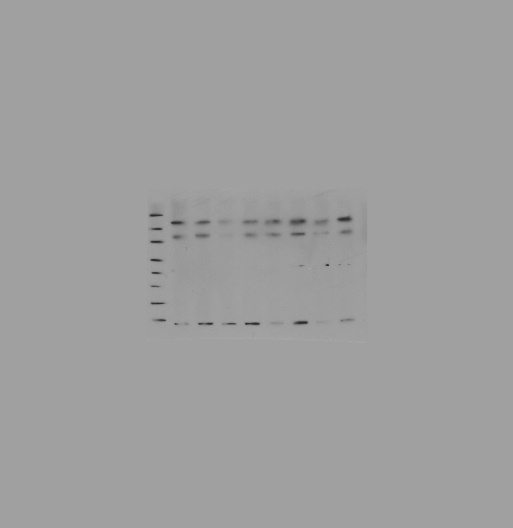

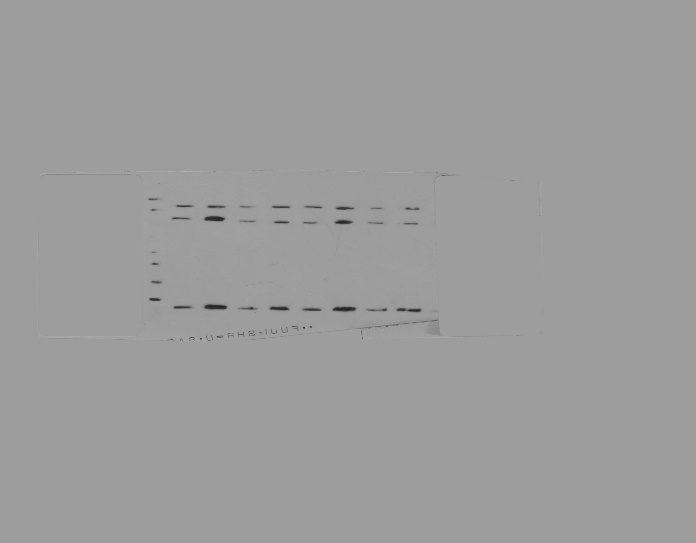


Figure S6. Original immunoblots images of **B-Amyloid in males and females for Figure 5**. Note. all blot membranes were cut into stripes prior to hybridization with antibodies. Membrane edges are not clear in some cases, due to high signal-to-noise ratio of luminescence intensity.

B-Amyloid

B-Amyloid

B-Amyloid

B-Amyloid

B-Amyloid

CTL Db Ex Db/Ex

CTL Db Ex Db/Ex

CTL Db Ex Db/Ex

CTL Db Ex Db/Ex

CTL Db Ex Db/Ex

CTL Db Ex Db/Ex

CTL Db Ex Db/Ex

CTL Db Ex Db/Ex

CTL Db Ex Db/Ex

CTL Db Ex Db/Ex

Female sample

Male sample

Male sample

Female sample

Male sample

Female sample

Male sample

Female sample

Male sample

Female sample

Male sample

Female sample

Β-Amyloid

84 KDa

CTL Db Ex Db/Ex

CTL Db Ex Db/Ex

Female sample

Selected sample for manuscript

B-Amyloid

CTL Db Ex Db/Ex

CTL Db Ex Db/Ex

Male sample


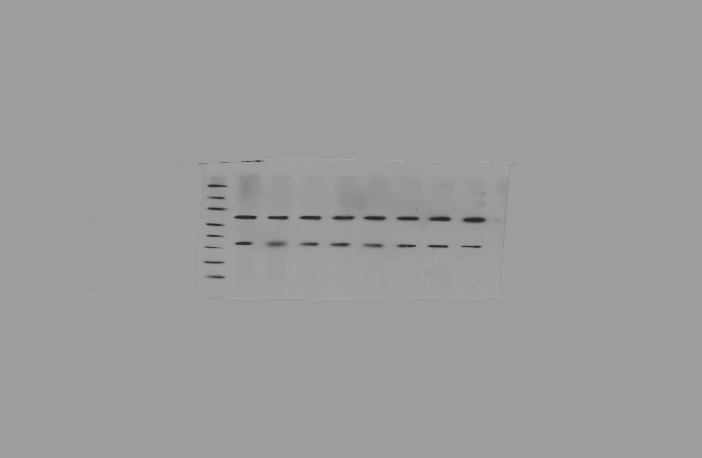

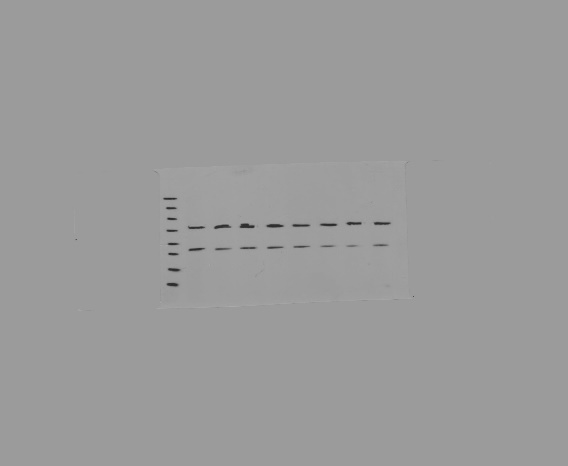

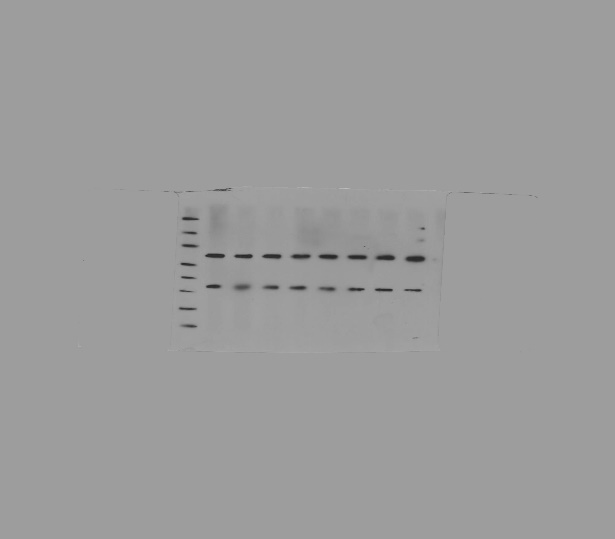


Tau

Tau

Tau

Tau

CTL Db Ex Db/Ex

CTL Db Ex Db/Ex

CTL Db Ex Db/Ex

CTL Db Ex Db/Ex

CTL Db Ex Db/Ex

CTL Db Ex Db/Ex

CTL Db Ex Db/Ex

CTL Db Ex Db/Ex

CTL Db Ex Db/Ex

CTL Db Ex Db/Ex

Female sample

Male sample

Female sample

Male sample

Female sample

Male sample

Female sample

Male sample

Female sample

Male sample

Female sample

Male sample

Tau

65 KDa

Selected sample for manuscript


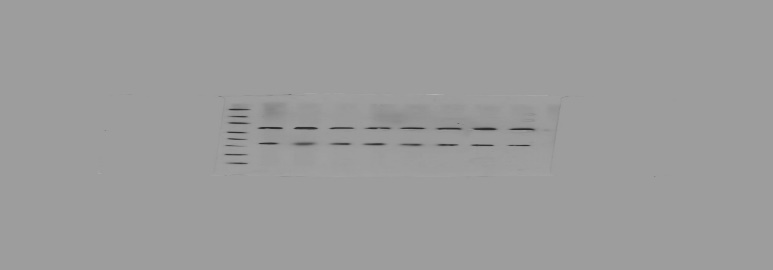

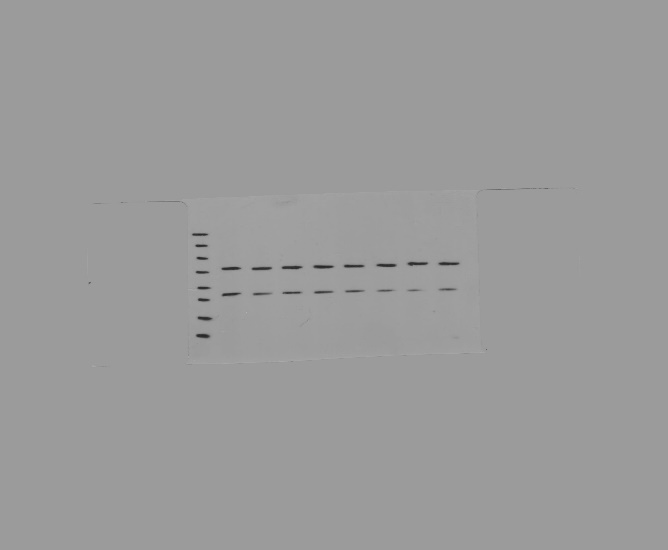

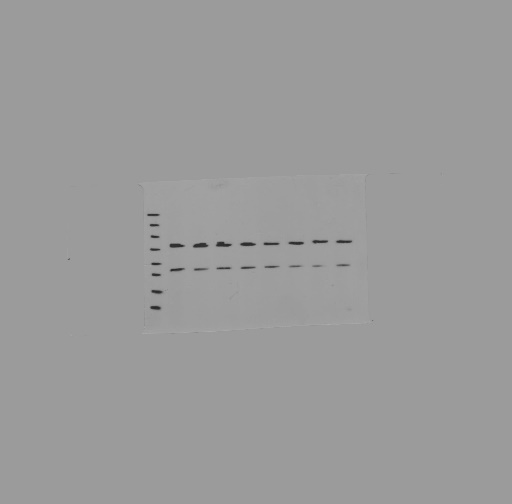

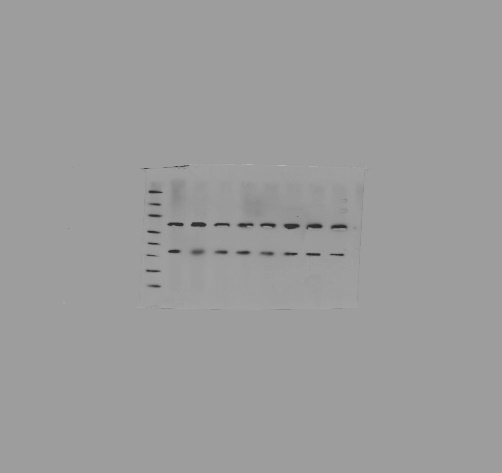


Tau

Tau

Figure S7. Original immunoblots images of **Tau in males and females for Figure 5**. Note. all blot membranes were cut into stripes prior to hybridization with antibodies. Membrane edges are not clear in some cases, due to high signal-to-noise ratio of luminescence intensity.

CTL Db Ex Db/Ex

CTL Db Ex Db/Ex

CTL Db Ex Db/Ex

CTL Db Ex Db/Ex

Female sample

Male sample


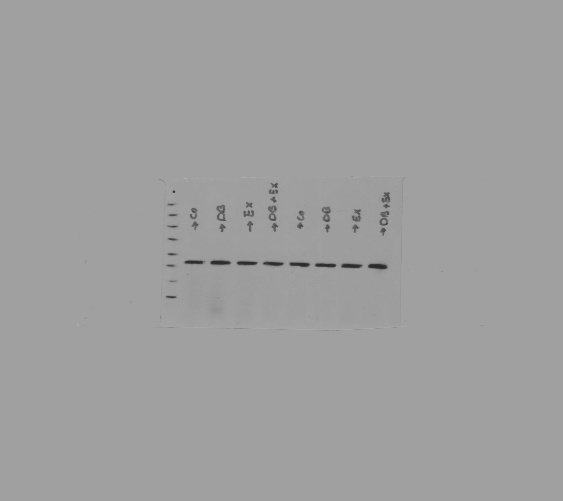

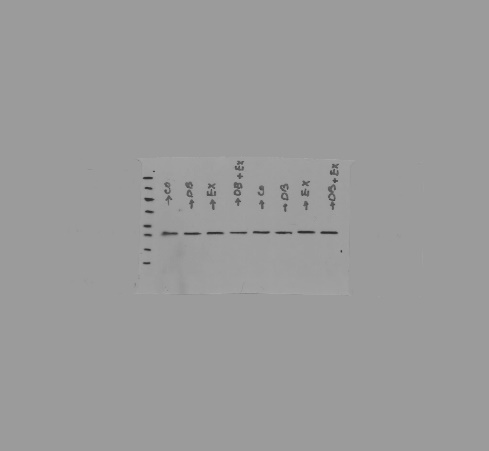


β Actine (42 KDa)

**β Actine**


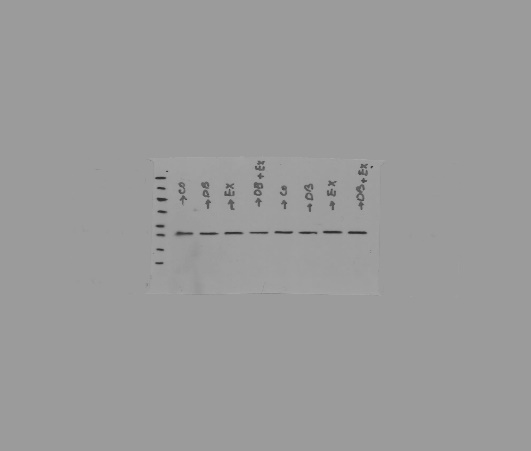

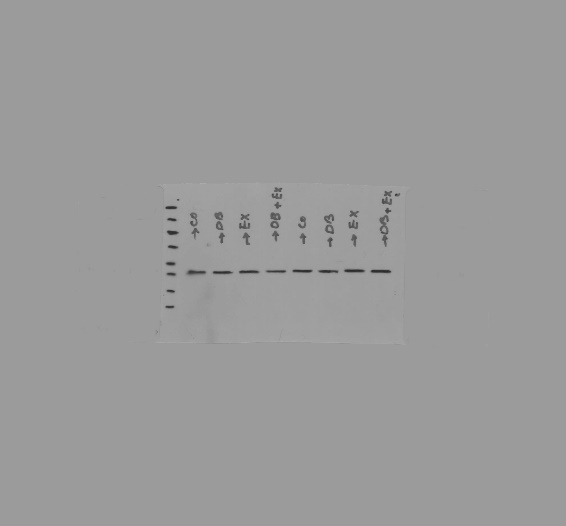


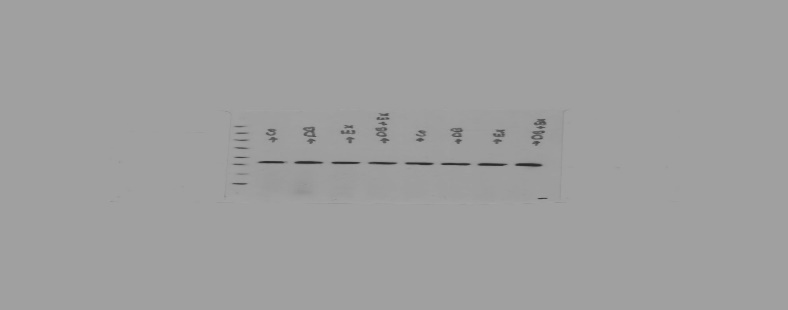

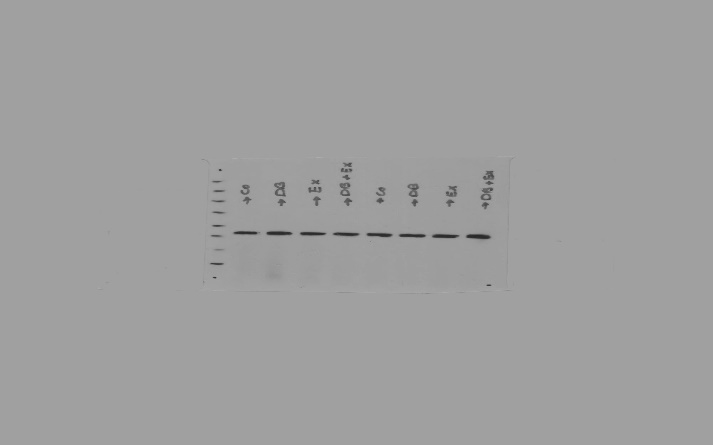


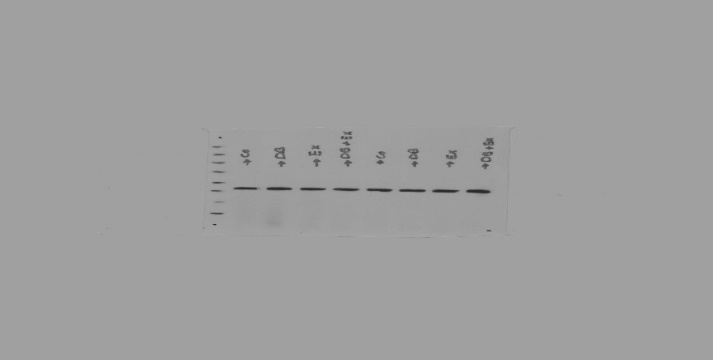


Figure S8. Original immunoblots images **for β Actine in males and females.** Note. all blot membranes were cut into stripes prior to hybridization with antibodies. Membrane edges are not clear in some cases, due to high signal-to-noise ratio of luminescence intensity
